# Impact of warming and reduced precipitation on morphology and chlorophyll concentration in peat mosses (*Sphagnum angustifolium* and *S. fallax*)

**DOI:** 10.1038/s41598-020-65032-x

**Published:** 2020-05-25

**Authors:** Anshu Rastogi, Michal Antala, Maciej Gąbka, Stanisław Rosadziński, Marcin Stróżecki, Marian Brestic, Radosław Juszczak

**Affiliations:** 1 Laboratory of Bioclimatology, Department of Ecology and Environmental Protection, Poznan University of Life Sciences, Piątkowska 94, 60-649 Poznan, Poland; 2 Department of Hydrobiology, Adam Mickiewicz University in Poznań, Uniwersytetu Poznańskiego 6, 61-614 Poznań, Poland; 3 Adam Mickiewicz University in Poznań, Uniwersytetu Poznańskiego 6, 61-614 Poznań, Poland; 4 Department of Plant Physiology, Slovak University of Agriculture, A. Hlinku 2, 94976 Nitra, Slovak Republic; 5 Department of Botany and Plant Physiology, Faculty of Agrobiology, Food and Natural Resources Czech University of Life Sciences, 16500 Prague, Czech Republic

**Keywords:** Projection and prediction, Environmental impact

## Abstract

Peatlands are one of the most important ecosystems due to their biodiversity and abundant organic compounds; therefore, it is important to observe how different plant species in peatlands react to changing environmental conditions. *Sphagnum* spp. are the main component of peatlands and are considered as the creator of conditions favorable for carbon storage in the form of peat. *Sphagnum angustifolium* and *Sphagnum fallax* are taxonomically very close species. To examine their adaptability to climate change, we studied the morphology and pigment content of these two species from environmental manipulation sites in Poland, where the environment was continuously manipulated for temperature and precipitation. The warming of peat was induced by using infrared heaters, whereas total precipitation was reduced by a curtain that cuts the nighttime precipitation. Morphology of *S. angustifolium* stayed under climate manipulation relatively stable. However, the main morphological parameters of *S. fallax* were significantly affected by precipitation reduction. Thus, this study indicates *S. angustifolium* is better adapted in comparison to *S. fallax* for drier and warmer conditions.

## Introduction

Climate change is appearing to be the biggest problem of this century. It is estimated that anthropogenic activities have caused global warming by approximately 1.0 °C in comparison with the pre-industrial era. The global warming rate is currently estimated to be 0.2 °C per decade; therefore, it is a high probability that the average global temperature may reach 1.5 °C higher in between 2032 and 2050^1^. Rainfall patterns affect surface wetness and water availability to plants; therefore, its alteration may directly impact the primary productivity^2^. In this changing environmental condition, plants may either adapt, move to suitable climatic conditions, or may get extinct^3^. With the change in plant species composition, the ecosystem may also lose its identity and can turn into a different ecosystem with changing environmental conditions^4^. Therefore, to understand the faith of the ecosystem in changing environmental conditions, it is crucial to study the behavior of different plant species.

Due to their high carbon storage capacity, peatlands are considered to be one of the most important ecosystems^5^. Peatlands cover only around 4 million km^2^ what amounts to 3% of the world’s terrestrial area, but contains around one-third of the terrestrial carbon^6^. About 80% of the world’s peatlands occur in the northern hemisphere^7^, and they constitute about 515 000 km^2^ in Europe^6^. Due to their high carbon storage, peatlands have attracted a lot of attention to environmental scientists^8–10^. It is still uncertain that under new climatic conditions (due to warming and changed precipitation) pristine peatlands will work like carbon sink or they will become a source of carbon^8,9^. The peatland vegetation is considered to be diverse with very complex and nonlinear behavior. Recently it has been shown that each species in peatlands may behave differently to changed environmental conditions, where the new ecological condition in peatlands may suit one species for its better growth, whereas other plant species may get impacted adversely^10^.

*Sphagnum* spp. are the most important components of the northern peatlands^11^. They are the key regulators of peatlands ecosystems as they create acidic, nutrient-poor, cold, and anoxic conditions, in which just a few other species can grow^12,13^. The photosynthesis in *Sphagnum* occurs only in the top 10 mm of the plant, mostly in the capitulum, but the lower part remains still alive and holds the ability to create side shoot in case of a damaging apex^14^. *Sphagnum*, like all bryophytes, does not have roots and conducting tissues^15^. It can absorb water and nutrients through the plant’s surface^12^. Very efficient water conduction and retention are possible due to the arrangement of stems, branches, and leaves of *Sphagnum*. Moreover, capillary conductance is supported by large hyaline cells with pores, which occupy a substantial part of tissues, larger than alive chlorophyll-containing cells and by growth in closely packed clusters^16,17^. The *Sphagnum* spp., unlike vascular plants, are poikilohydric (lack cuticle and stomata) and belong to poikilochlorophyllous plants (desiccation results in the loss of chlorophyll); therefore, photosynthesis is dependent on water uptake by capillary action, precipitation, and stored water in the plant^18,19^. Thus, the *Sphagnum* spp. are considered to be sensitive to increasing temperature and drought stress^20,21^. Understanding how these mosses response to climate change is one of the challenges towards understanding biodiversity on peatlands. Recently it was shown that phenotypic plasticity occurs among dominant *Sphagnum* spp., which allows the plant community to cope up with the changing environmental condition^21^.

Two of the most common species for poor fen peatlands are *Sphagnum angustifolium* (fine bogmoss) and *Sphagnum fallax* (flat-topped bogmoss) which may quite often occur together^22^. The plants belong to section Cuspidata, the section with hollow species, which has developed a “drought avoidance” strategy to avoid desiccation through its effective water holding morphology^23–25^. Because of its similarity and importance as peatlands restoration species, *S. angustifolium* and *S. fallax* were selected for this study^26,27^, where both species were compared for morphological changes and their pigment content under different environmental manipulated conditions.

The plant samples were collected from Rzecin peatland in Poland, where a unique climate manipulation site occurs^10^. The microclimate there is manipulated since 2014. The plants were collected from the plots which were continuously manipulated for 4 years by effective methods to create conditions similar to the expected change in climatic conditions in the next decades. The aim of our study was to answer the following questions: (1) does warming and reduced precipitation affected the morphology and pigment concentration of *Sphagnum* species? (2) does one species of *Sphagnum* sustained better in changed environment conditions when compared with the other?

Through this study, we also tried to prove the hypothesis that with climate change one *Sphagnum* species will be replaced with other suitable *Sphagnum* species.

## Results

### Variability in micrometeorological data of manipulated plots

The experiment was conducted in Rzecin (52°45′43′′N16°18′35′E, 54 m a.s.l.) peatland area in Poland. The microclimate of the site was continuously manipulated from 2014 to 2018^10,28–31^. Temperature and precipitation were recorded continuously at each of the four experimental sites marked as Control (C), Warming (W), Warming and Reduced Precipitation (WRP) and Reduced Precipitation (RP).

**Figure 1:**
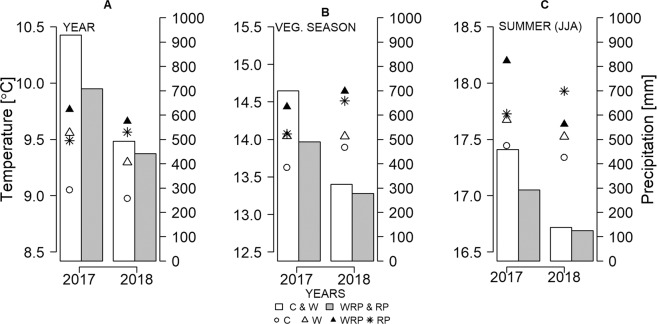
Mean temperature of peat measured at 50 mm depth and the amount of precipitation at control (C), warming (W), warming and reduced precipitation (WRP), and reduced precipitation (RP) sites of WETMAN climate manipulation experiment at Rzecin peatland for whole year (**A**), vegetation season from April till October (**B**) and summer from June till August (**C**). Warming was induced by the use of infrared heaters while precipitation was reduced by the curtain, which cut the amount of nighttime precipitation. Due to system failure, there was minimal precipitation reduction in 2018.

We observed a substantial variability of precipitation between the years. The year of 2017 was observed with the highest precipitation (Fig. 1A) sum of 929 mm, whereas 2015 was the driest among the studied years with a yearly sum of 417 mm^29^. The reduction in precipitation due to manipulation (in RP and WRP sites) was ca. 37% in years 2015–2016^29–31^, while in 2017 and 2018 it was smaller – 24% and 10% respectively, in comparison to the sites without a reduction in precipitation (C and W). Low precipitation reduction in the years 2017 and 2018 was caused due to the failure of the automated curtain system by a very strong storm in July 2017.

The average mean temperature of the peat was measured from 50 mm depth. The average annual peat temperature from all manipulated plots was observed to be higher on average by ca. 1.0 °C in the dry years 2015–2016^29–31^ and 0.9 °C in very wet 2017, in comparison to C plots. The year of 2018 was exceptional, where the increase in peat temperature was only around 0.2 °C for W plots, whereas for RP plots the increase was around 0.6 °C in comparison to C plots (Fig. 1A). The increase in average peat temperature was the highest for WRP sites and ranged from 0.7 to 0.8 °C comparing to C in 2017–2018.

As vegetation period or summer plays an important role in the plant life cycle, we have drawn the graph presenting changes in temperature and precipitation during vegetation period (April till October; Fig. 1B) and summer (June till August; Fig. 1C). Precipitation during vegetation period was observed to be reduced by 30% and 12% in 2017 and 2018, respectively, whereas, the summer precipitation was observed to be reduced by 36% and 10% for the years 2017 and 2018, respectively. During the studied period the difference in average peat temperature, in relation to C, for W site was observed to be between 0.2–0.6 °C during the vegetation period, whereas it was 0.2–0.3 °C for summer. The differences in average mean peat temperature for RP sites was 0.4–0.7 °C and 0.3–0.6 °C for WRP sites and 0.8–1.4 °C and 0.3–1.1 °C for the vegetation period and summer, respectively.

### Impact of warming and reduced precipitation on the morphology of *Sphagnum*

Sixteen different morphological traits related to plant length, capitulum, and branch length were measured. As the peatlands show dynamic and nonlinear behavior, the obtained data were analyzed in two ways, where the plant species from plots were separately analyzed by general linear mixed-effect models (GLMM) and ANOVA to observe the differences (Supplement Tables S1–3). Data were analyzed for significant differences between sites (C, W, WRP and RP) for every trait and every species separately (Tables 1–2). No significant changes between sites were observed in the length of whole plant and its green part for *S. angustifolium*, whereas the length of the brown part was significantly lower in W (Table 1). Dry mass of plant as well capitulum dry mass and its diameter in manipulated sites were not significantly different from C (Table 1).

**Table 1:** Average, minimal and maximal values of *Sphagnum angustifolium* traits in control (C), warming (W), warming and reduced precipitation (WRP) and reduced precipitation (RP) conditions.

Trait [unit]	C		W		WRP		RP	
	Mean	Min.-Max.	Mean	Min.-Max.	Mean	Min.-Max.	Mean	Min.-Max.
length of whole plant [mm]	88^a^	57–144	72^a^	53–94	93^a^	63–174	89^a^	49–199
length of green part [mm]	33^a^	20–51	41^a^	22–55	40^a^	19–64	36^a^	8–56
length of brown part [mm]	55^ac^	17–105	31^b^	9–54	54^ac^	0–116	52^abc^	12–143
length of green/brown part	0.81^ac^	0.19–2.55	1.84^b^	0.45.6.11	0.83^ac^	0.24–1.61	1.04^abc^	0.20–3.92
diameter of capitulum [mm]	12.7^a^	6.2-21-2	10^a^	5.6–17.8	12.5^a^	7.3–18.8	12.5^a^	7.9–19.5
dry mass of whole plant [g]	0.019^a^	0.007–0.031	0.027^a^	0.007–0.054	0.026^a^	0.006–0.053	0.022^a^	0.006–0.045
dry mass of capitulum [g]	0.008^ab^	0.003–0.014	0.011^ab^	0.002–0.024	0.012^ab^	0.004–0.023	0.006^a^	0.001–0.016
length of 1-st spreading branch [mm]	8.9^ab^	4.1–11.6	8.9^ab^	5.2–17.7	11.6^c^	7.5–16.9	10.4^abc^	4.7–15.6
length of 2-nd spreading branch [mm]	9.3^ab^	5.5–11.9	8.7^ab^	5.5–13.6	12.1^c^	7.2–17.3	10.8^abc^	7.2–15.1
length of 3-rd spreading branch [mm]	9.5^a^	7.2–13.3	9.4^a^	5.4–16.7	11.3^a^	6.7–16.2	10.6^a^	6.6–13.7
length of 1-st hanging branch [mm]	12.6^a^	6.7–18.4	11.6^a^	5.9–16.7	14.7^a^	8.8–22.5	12.9^a^	4.3–13.7
length of 2-nd hanging branch [mm]	12.3^ab^	8.1–16.9	13.1^abc^	6.7–19.7	15.6^bc^	9.2–23.4	13.1^abc^	8.5–16.5
length of 3-rd hanging branch [mm]	11.7^ab^	6.7–15.5	12.9^abc^	7.2–18.0	14.8^bc^	9.6–21.9	13.5^abc^	9.2–17.0
length of 1-st internodium [mm]	2.6^a^	1.2–4.9	2.8^a^	1.2–6.6	2.1^a^	0.6–3.4	2^a^	0.5–4.4
length of 2-nd internodium [mm]	2.7^a^	0.9–6.1	3.2^a^	0.6–6.1	2.2^a^	0.4–4.3	2.2^a^	1.0–5.6
length of 3-rd internodium [mm]	3.2^a^	1.5–8.1	3.9^a^	1.4–7.8	2.7^a^	0.9–7.9	2.2^a^	0.7–4.2
total chlorophyll content [mg.l^−1^]	12.3^a^	6.0–23.9	9.9^a^	4.6–15.6	12.5^a^	5.5–18.7	10.0^a^	4.9–15.6
chlorophyll a [mg.l^−1^]	7.8^a^	3.8–14.8	6.3^a^	3.0–10.1	8.0^a^	3.6–12.4	4.9^a^	3.1–6.3
chlorophyll b [mg.l^−1^]	4.5^a^	2.2–9.1	3.5^a^	1.6–5.5	4.4^a^	1.9–6.7	3.6^a^	1.8–5.5
chl a/chl b	1.7^a^	1.3–2.0	1.8^a^	1.6–1.8	1.8^a^	1.6–2.0	1.5^a^	0.7–1.91.6
carotenoids [mg.l^−1^]	2.6^a^	1.3–4.9	2.4^a^	1.2–3.7	2.7^a^	1.7–3.2	2.1^a^	1.0–3.5

Mean values with different letters (a–c) within each of the traits are significantly different from each other (p < 0.05).

**Table 2:** Average, minimal and maximal values of *Sphagnum fallax* traits in control (C), warming (W), warming and reduced precipitation (WRP) and reduced precipitation (RP) conditions.

Trait [unit]	C		W		WRP		RP	
	Mean	Min.-Max.	Mean	Min.-Max.	Mean	Min.-Max.	Mean	Min.-Max.
length of whole plant [mm]	81^a^	47–140	74^ab^	49–124	44^c^	24–81	54^bc^	31–99
length of green part [mm]	44^abc^	17–89	50^ab^	16–72	33^ac^	24–45	30^ac^	14–53
length of brown part [mm]	37^ab^	13–75	24^abc^	0–63	10^bc^	0–45	24^abc^	0–56
length of green/brown part	1.60^a^	0.36–5.80	2.33^a^	0.31–5.89	2.49^a^	0.80–6.40	2.10^a^	0.44–5.80
diameter of capitulum [mm]	13.0^a^	6.6–20.1	9.1^b^	6.8–13.6	6.8^b^	4.9–8.9	8.0^b^	3.8–16.6
dry mass of whole plant [g]	0.024^a^	0.005–0.074	0.026^a^	0.013–0.040	0.009^a^	0.003–0.014	0.009^a^	0.005–0.015
dry mass of capitulum [g]	0.007^ac^	0.002–0.017	0.011^bc^	0.005–0.021	0.005^abc^	0.002–0.008	0.003^ac^	0.002–0.005
length of 1-st spreading branch [mm]	10.3^a^	4.4–15.5	10.3^a^	5.8–14.4	11.3^a^	6.4–18.7	9.0^a^	7.6–12.3
length of 2-nd spreading branch [mm]	10.6^a^	4.6–17.9	9.7^a^	5.9–14.7	10.6^a^	7.8–15.9	9.7^a^	6.4–13.7
length of 3-rd spreading branch [mm]	10.7^a^	5.1–19.0	11.1^a^	7.0–17.4	10.6^a^	5.5–15.3	10.5^a^	6.7–14.2
length of 1-st hanging branch [mm]	12.8^a^	6.4–19.3	12.4^a^	8.3–16.9	12.5^a^	5.7–16.9	10.5^a^	7.0–13.8
length of 2-nd hanging branch [mm]	12.5^a^	7.8–20.0	12.5^a^	6.7–15.8	10.5^a^	5.4–13.6	10.5^a^	7.0–12.2
length of 3-rd hanging branch [mm]	13.6^a^	6.5–19.7	12.9^a^	7.2–18.5	12.4^a^	6.8–17.7	10.5^a^	6.1–14.6
length of 1-st internodium [mm]	2.8^a^	1.0–3.9	2.9^a^	0.7–7.2	1.8^a^	0.7–3.2	2.1^a^	1.0–3.4
length of 2-nd internodium [mm]	3.4^a^	1.3–6.8	2.9^a^	0.4–6.8	2.2^a^	1.1–4.2	2.6^a^	1.1–6.6
length of 3-rd internodium [mm]	3.7^a^	1.4–5.6	3.1^a^	1.6–5.2	3.1^a^	1.5–7.4	2.9^a^	0.8–5.4
total chlorophyll content [mg.l^−1^]	9.1^a^	4.8–13.1	10.7^a^	7.3–17.5	9.7^a^	6.1–13.5	7.9^a^	3.9–11.2
chlorophyll a [mg.l^−1^]	5.8^a^	2.9–9.0	7.0^a^	4.5–12.0	6.3^a^	3.7–8.9	5.1^a^	2.5–7.5
chlorophyll b [mg.l^−1^]	3.6^a^	1.9–5.5	3.7^a^	2.8–5.5	3.4^a^	2.4–4.5	2.8^a^	1.5–3.7
chl a/chl b	1.6^a^	1.1–1.9	1.8^a^	1.6–2.2	1.8^a^	1.6–2.1	1.8^a^	1.7–2.1
carotenoids [mg.l^−1^]	2.1^a^	1.2–3.0	2.9^a^	1.7–4.8	2.3^a^	1.7–2.9	1.9^a^	1.2–2.4

Mean values with different letters (a–c) within each of the traits are significantly different from each other (p < 0.05).

In the case of *S. fallax*, very important morphological traits like length of whole plant, its green or brown part, diameter and dry mass of capitulum showed significant differences between sites (Table 2). The highest values of parameters total length, length of brown part and capitulum diameter were observed in the C. The lowest values of length of plant and its parts, capitulum diameter and its dry mass were recorded in sites with manipulated precipitation (Table 2).

### Impact of warming and reduced precipitation on pigments of *Sphagnum*

The concentration of chlorophyll-a (chl a), chlorophyll-b (chl b), and carotenoids were observed from both the plant species from different experimental sites. In *S. angustifolium* all the observed pigments were higher in comparison to *S. fallax*. A decrease in all the studied pigments was observed for the RP sites for both the studied species in comparison to C. Carotenoid content was higher for W and WRP for *S. fallax*, whereas it was observed to be relatively stable for *S. angustifolium*. The ratio of chl a and chl b was observed to be slightly higher for both the studied plants in W and WRP sites in comparison to C.

## Discussion and Conclusions

Dominating plant species plays an important role in ecosystem sustainability. With the change in environment, the conditions in the ecosystem may change which may favor one species more than others. The new favorable species may accelerate the process of change in microhabitat, which may cause the transformation of the ecosystem. Therefore, the study related to species response to changing environmental conditions is important. In this study, we have shown how the two very similar and important plant species of peatlands reacted to the environmental change through climate manipulation experiments. *S. angustifolium* and *S. fallax* are very similar species from section Cuspidata. They are commonly found to coexist together, like in the Rzecin peatland area in Poland. *Sphagnum* spp. are considered to be sensitive towards water availability, change in water conductivity, and pH due to precipitation or temperature change^20,32^. *S. fallax* can grow under more acidic condition (i.e. from 3.1 to 6.1 pH) in comparsion to *S. angustifolium* (3.8 to 7 pH). Therefore, alteration in precipitation or temperature can alter the *Sphagnum* community in peatlands. A recent study has shown that the *Sphagnum* community declined under warmer conditions but has considered *S. angustifolium* and *S. fallax* together assuming that they have similar responses to change in environmental conditions^20^, whereas in this study, we have observed that *S. angustifolium* and *S. fallax* reacted differently to warming and reduced precipitation. The different responses of plant species were analyzed through the observation of plant morphology and pigment composition after four years of continuous exposure of plants to manipulated conditions, and their comparison to the observations from the plants without manipulations.

Several authors^23,33,34^ reported a significant increase in plant length of *Sphagnum* spp. under warmer conditions when compared with control, whereas, we observed a slight decrease in total plant length for both the plant species for W. Interestingly plants from WRP and RP showed the opposite response to manipulation for both the studied plants, where *S. angustifolium* was observed to be slightly longer and *S. fallax* was observed to be significantly shorter in comparison to C. Length of green and brown part almost followed the similar pattern except for W, where the length of green part was observed to be slightly longer in comparison to control. The longer green part in W can be the influence of better light penetration which may be higher in W because of the compactness of capitulum, which can be seen by lower diameter and higher dry mass capitulum in W. The observation clearly indicated that the growth of studied species was strongly correlated with precipitation in comparison to temperature, which was in agreement with the previous study^35^. Further studies should corroborate if this is a common feature throughout the different ecoregions.

Most of the light absorption and CO_2_ assimilation occurs in the topmost part of *Sphagnum* plants, which is known as capitulum^36^. The warming of the environment may induce the growth of *Sphagnum* and stimulate dry matter production as long as the water availability is sufficient^36^. But higher temperatures may lead to higher evapotranspiration, which makes the peat dry. Therefore, the capitulum of *Sphagnum* gets smaller, a similar response was observed in our study. The water loss by evapotranspiration must be compensated by rain or capillary uptake from deeper peat layers^37^. The water loss from peatlands due to evapotranspiration cannot be restored by rainwater in 100%^36^. The increased temperature was previously observed to increases capillary water flow and evapotranspiration ratio^38^. *Sphagnum* in dry conditions enters a state of desiccation, which significantly reduces evapotranspiration and photosynthesis^39^. If the plant capitulum is dry for a long time, less photosynthesis occurs which results in the reduction of plant growth. Therefore, the water content of capitulum is decisive for the growth of *Sphagnum*^35,36,40^. Shorter *S. fallax* plants with smaller capitulum in RP and WRP conditions in comparison with C shows a negative influence of reduction in precipitation which indicates that the capillary uptake of water in *S. fallax* may probably not replace the loss of water due to evapotranspiration which results in poor growth of the plant. The capitula layer of *Sphagnum* controls evaporation and water retention in the plant^39^. Although the capitulum diameter differs between experimental sites, the differences were significant (p < 0.05) just for *S. fallax*. As the reduced precipitation sites showed lower capitulum diameter for *S. fallax*, we can conclude that the capitulum diameter of *S. fallax* was depended on the precipitation.

The decrease in total dry mass of plant and dry mass of capitulum in W and WRP for *S. fallax* indicate the importance of water in dry mass production of mosses reported earlier^41^. The observation indicated *S. fallax* to be strongly influenced by reduced precipitation in comparison to *S. angustifolium*.

The shortest brown part (i.e. decaying part) was observed in WRP for *S. fallax*, whereas it was in W for *S. angustifolium*. The observation indicated that the best conditions for the decomposition of *S. fallax* peat were in WRP probably caused by better conditions for decomposing bacteria and saprotrophic fungi^42,43^. The observation is in agreement with the previous study where warmer and drier conditions have been reported to cause a 50% decrease in *S. fallax* occurrence and substantial reduction of productivity and photosynthetic activity with a significant increase in peat decomposition^44^.

*S. fallax* in C was observed to have a slightly bigger capitulum and longer plant then *S. angustifolium*. The observation indicates that the normal environmental condition was suitable for the coexistence of both the species, where both species may have long and healthy plants. Similar behavior of both species was observed from some random sites away from the experimental site (but still on floating peatland area), where both species were observed to be of comparable length (data not shown). But the longer *S. angustifolium* in comparison to *S. fallax* in manipulated sites indicates the manipulation negatively influenced the growth of *S. fallax*. *S. fallax* is considered to be having better productivity under acidic growth condition^38^, but the pH observation of water from different sites does not show any differences (data not shown). *S. angustifolium* is the moss considered to be growing better at an open area, which was the case of our manipulated site^45^. Good growth of *S. angustifolium* in comparison to other lawn *Sphagnum* spp. has been observed by different authors^46^. In a study the multispecies comparison performed during two contrasting years, wet and dry, showed higher biomass increment for *S. angustifolium* in the wet year^23^. The reduced rainfall during vegetation season was enough for the normal growth of *S. angustifolium*, which is visible on similar lengths of the plant and also capitulum diameter. However, shorter plants and smaller capitulum of *S. fallax* in RP and WRP conditions showed that reduced precipitation was not enough for their normal growth. It may be explained by that, if the drought lasts too long the *Sphagnum* water content drops under-compensation point, which causes irreversible damage^40^. The compensation point of *S. fallax* seems to be higher than that of *S. angustifolium*; therefore, *S. angustifolium* can better use accessible water after a period of dryness.

We collected only the wet and green *Sphagnum* from each plot; therefore, we can consider, all measured plants of both species in all plots were alive and photosynthetically active. Generally, under stress conditions, the chlorophyll content is lower in comparison to healthy plants^47^, but as the peatlands environment is water-saturated, and the *Sphagnum* can uptake the water through capillary action, the plants are able to maintain their photosynthetic activity. Warming may even induce the activity of certain enzymes involved in chlorophyll production, especially at morning hours when sunlight is sufficient, this may impact the total chlorophyll content positively. Chl a is the main photosynthetic pigment that forms core antenna molecules, whereas chl b are accessory molecules that participate in light harvesting^48^. In *S. angustifolium*, the total chlorophyll concentration was observed to be higher, which indicates a better photosynthetic condition for *S. angustifolium* when compared with *S. fallax*. Generally, the chl a/b ratio in the healthy plant is around 3, but in our observation, we observed the ratio to be around 2, which indicates in general photosynthetic apparatus was not intact in this period, which was in agreement with the previous observation on S. *lindbergi*^12^. An improvement in chl a/b ratio at the manipulated condition for both plant species indicated a better status of photosynthetic apparatus in manipulated conditions during the measurement period. Carotenoids are other important molecules in photosynthesis which capture the blue-green light and transfer it to chlorophyll^49^. There was a slight change in carotenoid concentration when compared between different experimental sites, but no significant differences were observed between the species.

Our results provide information that how the changes in precipitation may impact the *Sphagnum* population in northern peatlands. Different climate model indicates a sharp shift in precipitation where climate change may lead to a long drier period in northern region^50^. *S. fallax* is one of the most common *Sphagnum* mosses in European peat bogs^51^. Despite the fact that *S. fallax* prefers drier and nutrient richer habitats in comparison to *S. angustifolium*^52^, its growth was observed to be more dependent on precipitation, which is indicated by shorter plants of this species with smaller and lighter capitulum. These show the less adaptive capacity of *S. fallax* to studied manipulated conditions. The observation clearly indicated that among the two studied species *S. angustifolium* has better potential to survive in the climatic conditions where dry periods will be longer. Our results suggested that due to increased temperature and reduced availably of water, *S. fallax* can be substituted by another closely related species like *S. angustifolium* in conditions like northern Poland. The substitution can be due to better growth of *S. angustifolium*, which may outcompete the *S. fallax*. The shift in *Sphagnum* community composition is usually connected with the substitution of hollow species by hummock species^20,36,38^. Whereas, through this study, we have shown that the shift may also occur from one hollow species to another. Thus the study is important to understand how *Sphagnum* community may maintain ecosystem functioning when subject to future climatic condition. The shift in *Sphagnum* spp. is required to maintain the total peatland productivity and helps to maintain the carbon balance of peatland. The study is also important for peatland conservation as it helps to understand the behavior of two related *Sphagnum* spp.

## Material and Methods

### Study site

Plants for this study originated from experimental site WETMAN established in 2014 at Rzecin peatland (52°45′43′′N16°18′35′E, 54 m a.s.l.) in Greater Poland region, north-western Poland^53–55^. Rzecin is a large complex of peatland (86 ha) with dominated mesotrophic fen. The fens are overgrown by vascular plants and mosses, and makes a floating peat-substrate carpet of 500–700 mm in the middle of the peatland. The experimental site is situated on the floating part of the peatland which was examined to be similar type of vegetation distribution in the year 2014. This fen has considerable biodiversity and possesses many rare plant species (e.g., *Liparis loeselii, Carex dioica*, *Hamatocaulis vernicosus*, *Cinclidium stygium, Paludella squarrosa* and *Helodium blandowii*). Dominant species of vegetation are *Sphagnum* spp. (in summary 11 taxa of *Sphagnum*), *Carex rostrata* (*C*. *rostrata*), *C. lasiocarpa, C. limosa, Calamagrostis stricta*, *Phragmites australis*, *Typha latifolia*, *Oxycoccus palustris*, *Drosera rotundifolia*, *Ranunculus lingua*, and *Menyanthes trifoliata*. According to FAO 2006 classification, the soil substrate is a Limnic Hemic Floatic Ombric Rheic Histosol (Epidystric)^53,54^.

**Figure 2:**
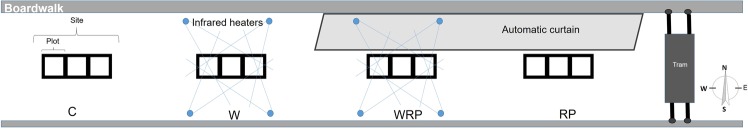
The illustrative diagram of the WETMAN climate manipulation experimental sites, showing four experimental conditions: control (C), warming (W), warming and reduced precipitation (WRP), and reduced precipitation (RP). The illustration also shows the positioning of infrared heaters and an automatic curtain. A rope driven tram is shown, which was used for passing over the plots for the purpose to collect samples.

WETMAN climate manipulation experiment consists of 4 experimental plots covering 2.5 × 2.5 m of peatland (Fig. 2). The experimental sites are control (C), warming (W), reduced precipitation (RP), and combination of warming with reduced precipitation (WRP); within each of them, there are three experimental plots of size 0.7 × 0.7 m as a main long-term experiment. The increase in temperature was achieved by installing four 400 W infrared radiators per W and WRP sites, while for reducing precipitation the automatic curtain covering at RP and WRP sites during the night was used. For the purpose to minimize the cost of heating the experimental plots are grouped together. In previous works the experimental setup and manipulation methods are described in detail^10,28,55^.

### Temperature and precipitation measurements

Peat temperature was measured continuously in each of the twelve plots at 50 mm depth by using T-107 thermistors (Campbell Sci. USA). Precipitation was measured with the four-headed TPG-124-H24 rain gauges (ASTER, Poland) with two sensors exposed to rain-manipulated and two to non-rain-manipulated conditions.

### Species identification and sampling

Live *Sphagnum* species were identified on-site using a microscope Zeiss Axioplan 2 (Carl Zeiss Microscopy, LLC, Thornwood, USA) and magnifiers with a magnification of x20 (Lichen candelaris x20). During identification, attention was paid to the most important features distinguishing two closely related *Sphagnum* species (Table 3). *Sphagnum angustifolium* (Warnst.) C.E.O. Jense has red branch-bases and a partially pure red stem, while *Sphagnum fallax* (Klinggr.) Klinggr. has a pale, green, or yellow stem and pale green or yellowish branches^56^. Moreover, *S. angustifolium* has shorter stem leaves than the type of *S. fallax*, and their apex is flattened and usually torned.

**Table 3:** Identification of species – most important macroscopic features.

Species	Colour			Stem leaves					Capitulum	Branch leaves
	Green	Partially pure red stem	Red branch-bases	Small <1 mm	Ovate triangular and broadest at base	Broadly equilateral triangular	Apex apiculate to acute	Obtuse apex	Capitulum hemispherical	In 5-ranked rows
*Sphagnum angustifolium*	+	+	+	+	−	+	−	+	+	−
*Sphagnum fallax*	+	−	−	(+)	+	−	+	−	−	+

(Key diagnostic features which distinguish of *Sphagnum angustifolium* and S. *fallax*. Symbol + means valid for species, (+) partially valid and − invalid.

Samples were collected from the study site twice during summer 2018. The first sampling was performed on 20^th^ July, and the second was done on 18^th^ August 2018. For sampling each site is divided into three zones which correspond to the plots. The main drawback of WETMAN experimental sites is that plots are grouped together. By considering the size of the plants and selection process for WETMAN experiment where the peatland was carefully selected for plants with a similar mix of different plants and growth conditions, we can consider each plot to be separate. Plant samples were taken from the outside edge (maximum 15 cm from the metal frame, for the purpose to not disturb ongoing long-term measurement of carbon fluxes due to plant alteration) of the manipulated region. A rope operated tram (shown in Fig. 2) was used to go over the plot and collect the samples. Samples for chlorophyll measurement were taken from 5 plants per experimental plot (top 30 mm shoot was taken for each) and were immediately stored in liquid nitrogen. For every sampling site (C, W, WRP and RP), at least three repeating samples consisted of 5 plants for each species were taken (in total, 15 plants of the same species were collected per site).

Plants for morphological measurements were separately packed in polybags and stored at 4 °C till measurements were performed at the laboratory.

### Morphological measurements

Dry mass of whole plant, dry mass of capitulum, length of whole plant, length of the green and brown part of plant, capitulum diameter, length of the first three internodien and length of the longest one spreading and hanging branch from each of the first three nodien were measured for at least 11 plants from each site. The length of the whole plant was measured as a distance between the top of the capitulum and the end of the stem connected with capitulum. Part of the plant from capitulum (capitulum including) to visibly green-brown color transition was considered as the green part of a plant. Part of the plant from green-brown transition point to lower end was considered as the brown part. Length of the whole plant, its green and brown parts were measured by caliper (Topex. Warsaw. Poland) with 1 mm accuracy and length of internodien, spreading and hanging branches and capitulum diameter were measured with 0.1 mm accuracy.

Samples for dry mass were dried in the oven at 64 °C until a constant weight was achieved. The dry mass of the whole plant and dry mass of capitulum were measured with 0.0001 g accuracy.

### Plant pigment measurements

Samples were crushed in liquid nitrogen in a mortar till smooth and dry powder was obtained after the evaporation of nitrogen. Immediately after, 5 ml of 80% acetone was added to the sample. The mixture was transferred to a test tube and centrifuged at 3000 RPM for 3 minutes by using centrifuge MPW-212 (MPW MED. INSTRUMENTS, Warsaw, Poland). The supernatant was immediately transferred to cuvette and absorbances at wavelengths 470 nm, 647 nm and 663 nm were measured by spectrophotometer SP-830 plus (Metertech Inc., Taipei. Taiwan). Chlorophyll and carotenoids contents were established according to the methodology established by Lichtenthaler^57^.

### Statistical analysis

The differences between sites (C, W, WRP, and RP) for every trait and every species separately were analyzed using general linear mixed-effect models (GLMM), with each site (C, W, WRP and RP) as a random factor^58^. One-way ANOVA with Turkey HSD test was performed to analyze significant (p < 0.05) differences for every trait and every species separately. All statistical analyses were carried out by using Statistica, ver. 12 (Statsoft Inc., Tulsa, OK, USA) and RStudio, ver. 2016 (RStudio Inc., Boston, MA, USA)^59^.

## Supplementary information

Supplementary tables.
