# Supplementary material for: Impact of warming and reduced precipitation on morphology and chlorophyll concentration in peat mosses (Sphagnum angustifolium and S. fallax)
Source: Sci Rep. 2020 May 25;10:8592. doi: 10.1038/s41598-020-65032-x (PMC7248058; doi:10.1038/s41598-020-65032-x)
Supplement: Supplementary file 1 — Supplementary tables. [file 41598_2020_65032_MOESM1_ESM.docx]

**Statistical evaluation of the studied plant traits**

**Impact of warming and reduced precipitation on morphology and chlorophyll concentration in peat mosses (*Sphagnum angustifolium* and *S. fallax*)**

**Table S1.** Results of the linear mixed-effect models of *Sphagnum angustifolium* features in experimental plots conditions (control, warming, reduced precipitation and warming and reduced precipitation). The mean difference is significant at the 0.05 level.

| **Trait** | F | p |
| --- | --- | --- |
| **Random factor** | 1.095 | 0.342 |
| **length of whole plant** | 2.062 | 0.097 |
| **length of green part** | 1.298 | 0.281 |
| **length of brown part** | **3.565** | **0.011** |
| **diameter of capitulum** | **2.529** | **0.050** |
| **length of 1-st spreading branch** | 1.077 | 0.376 |
| **length of 2-nd spreading branch** | 1.411 | 0.241 |
| **length of 3-rd spreading branch** | 1.720 | 0.158 |
| **length of 1-st hanging branch** | 2.469 | 0.054 |
| **length of 2-nd hanging branch** | **4.718** | **0.002** |
| **length of 3-rd hanging branch** | 1.723 | 0.157 |
| **length of 1-st internodium** | 1.826 | 0.136 |
| **length of 2-nd internodium** | **2.981** | **0.026** |
| **length of 3-rd internodium** | 2.363 | 0.063 |
| **dry mass of whole plant** | **4.199** | **0.004** |
| **dry mass of capitulum** | 1.716 | 0.155 |
| **chlorophyll a** | 1.207 | 0.336 |
| **chlorophyll b** | 1.212 | 0.334 |
| **total chlorophyll content** | 1.219 | 0.331 |
| **carotenoids** | 0.616 | 0.656 |

**Table S2.** Results of the linear mixed-effect models of *Sphagnum fallax* features in experimental plots conditions (control, warming, reduced precipitation and warming and reduced precipitation). The mean difference is significant at the 0.05 level.

| Trait | F | p |
| --- | --- | --- |
| **Random factor** | 2.731 | **0.002** |
| **length of whole plant** | 15.213 | **0.000** |
| **length of green part** | 18.047 | **0.000** |
| **length of brown part** | 4.502 | **0.004** |
| **diameter of capitulum** | 10.366 | **0.000** |
| **length of 1-st spreading branch** | 2.525 | 0.053 |
| **length of 2-nd spreading branch** | 1.009 | 0.412 |
| **length of 3-rd spreading branch** | 0.898 | 0.473 |
| **length of 1-st hanging branch** | 4.801 | **0.002** |
| **length of 2-nd hanging branch** | 4.679 | **0.003** |
| **length of 3-rd hanging branch** | 4.525 | **0.003** |
| **length of 1-st internodium** | 5.079 | **0.002** |
| **length of 2-nd internodium** | 10.457 | **0.000** |
| **length of 3-rd internodium** | 5.798 | **0.001** |
| **dry mass of whole plant** | 7.652 | **0.000** |
| **dry mass of capitulum** | 17.937 | **0.000** |
| **chlorophyll a** | 0.675 | 0.616 |
| **chlorophyll b** | 0.657 | 0.627 |
| **total chlorophyll content** | 0.665 | 0.622 |
| **carotenoids** | 1.768 | 0.168 |

**Table S3.** Degree of freedom (Df), F value and P value of analysis of variance among and between treatments for *S. angustifolium* and *S. fallax*.

P values in bold mean statistically significant differences between treatments at the level of α<0.05, * means statistically significant differences at the level of α<0.01 and ** means statistically significant differences at the level of α<0.001

| **Trait** | ***S. angustifolium*** | | | ***S. fallax*** | | |
| --- | --- | --- | --- | --- | --- | --- |
|  | **Df** | **F value** | **P value** | **Df** | **F value** | **P value** |
| **length of whole plant** | 3 | 2.155 | 0.103 | 3 | 6.36 | **<0.001**** |
| **length of green part** | 3 | 1.367 | 0.261 | 3 | 4.97 | **0.004*** |
| **length of brown part** | 3 | 3.333 | **0.025** | 3 | 4.484 | **0.007*** |
| **length of green/brown part** | 3 | 3.965 | **0.012** | 3 | 0.636 | 0.596 |
| **diameter of capitulum** | 3 | 1.494 | 0.225 | 3 | 14.09 | **<0.001**** |
| **dry mass of whole plant** | 3 | 2.586 | 0.059 | 3 | 2.098 | 0.113 |
| **dry mass of capitulum** | 3 | 4.703 | **0.005*** | 3 | 4.836 | **0.006*** |
| **length of 1-st spreading branch** | 3 | 2.471 | 0.070 | 3 | 1.580 | 0.206 |
| **length of 2-nd spreading branch** | 3 | 3.857 | **0.014** | 3 | 1.981 | 0.129 |
| **length of 3-rd spreading branch** | 3 | 3.203 | **0.030** | 3 | 2.335 | 0.085 |
| **length of 1-st hanging branch** | 3 | 3.317 | **0.026** | 3 | 1.458 | 0.237 |
| **length of 2-nd hanging branch** | 3 | 6.369 | **<0.001**** | 3 | 0.419 | 0.740 |
| **length of 3-rd hanging branch** | 3 | 4.284 | 0.088 | 3 | 0.083 | 0.969 |
| **length of 1-st internodium** | 3 | 1.429 | 0.243 | 3 | 3.188 | **0.031** |
| **length of 2-nd internodium** | 3 | 1.829 | 0.151 | 3 | 1.372 | 0.262 |
| **length of 3-rd internodium** | 3 | 2.330 | 0.083 | 3 | 0.852 | 0.472 |
| **total chlorophyll content** | 3 | 0.486 | 0.695 | 3 | 0.797 | 0.507 |
| **chlorophyll a** | 3 | 1.346 | 0.284 | 3 | 0.737 | 0.540 |
| **chlorophyll b** | 3 | 0.515 | 0.676 | 3 | 0.993 | 0.412 |
| **chl a/chl b** | 3 | 2.065 | 0.133 | 3 | 2.225 | 0.110 |
| **carotenoids** | 3 | 0.182 | 0.907 | 3 | 1.739 | 0.185 |
